# Computed tomographic features of the prostatic gland in neutered and intact dogs

**DOI:** 10.1186/s12917-020-02374-8

**Published:** 2020-05-24

**Authors:** N. Kuhnt, L. K. Harder, I. Nolte, P. Wefstaedt

**Affiliations:** Small Animal Clinic, University of Veterinary Medicine Hannover Foundation, Bünteweg 9, 30559 Hannover, Germany

**Keywords:** Canine prostate, Castrated dogs, CT, Hounsfield units, Tissue density

## Abstract

**Background:**

Aim was to investigate age-dependent changes in the prostate of castrated dogs in computed tomographic (CT) examination.

Thirty-six canine prostates were evaluated in pre- and post-contrast CT scans. Dogs were divided in groups with homogenous prostatic tissue (25/36) and with tissue alterations (11/36). Prostatic attenuation in Hounsfield Units (HU) and prostatic size were measured and a ratio of the prostatic size to the sixth lumbar vertebra was calculated. Additionally, the CT images of the prostate were compared with ultrasound examination.

**Results:**

In pre-contrast CT scans no significant differences were found in prostatic size between homogenous and altered prostatic tissue groups whereas prostatic attenuation differed significantly in post-contrast CT between these groups. The homogenous tissue pattern of homogeneous prostates could be confirmed in CT images and in ultrasound examination. Concerning prostates with alterations, the results differed between ultrasound and CT examination in four cases of 11 dogs with tissue alterations.

**Conclusions:**

CT is beneficial to examine the prostate of castrated dogs. The prostatic attenuation is characteristic for the prostatic morphology, which can vary due to ageing processes. Differences in attenuation and size can be found between prostates of castrated and intact dogs. Using contrast agent, CT can visualize prostatic alterations, which were not seen in ultrasound. The presented results should be considered preliminary until a study with larger sample size and histologic examination of the prostates is performed.

## Background

Computed tomography (CT) is based on X ray densitometry and enables an investigation of the prostate without superimposition by other anatomic structures. Furthermore, CT allows reconstructing images in different planes [1–3]. Thus, it is possible to display the prostate in transversal, sagittal and dorsal plane. In combination with contrast agent application it may give additional information about the vascular system which is especially valuable for tumor imaging [4]. Previous studies have evaluated the prostate of intact dogs using CT [5–7]. Lee et al. [5] examined the prostate of intact dogs in CT, which did not have any urological signs. The results of the CT measurement of the prostatic size had similar findings as those measured by sonographic examination. Classifying the intact male dogs in one group with healthy dogs and the second group in dogs with benign prostatic hyperplasia (BPH), Pasikowska et al. [6] found out that dogs with a healthy prostate had higher attenuation values than dogs with BPH. The prostatic size was smaller in dogs with a healthy prostate than in dogs with a BPH. Age- related morphological differences in the prostate of intact dogs were worked out in the study of Kuhnt et al. [7]. Pre- and post-contrast CT- examinations showed that most of the young dogs had a homogenous prostate while older dogs had cysts and inhomogeneous prostatic tissue. Measuring the radiographic attenuation of the prostatic tissue it was demonstrated that age-related changes and alterations were reflected in different Hounsfield Units (HU) as well. Atalan et al. [8] evaluated the prostatic size in canine cadavers of intact and castrated dogs. In intact dogs the prostatic weight and volume as well as the body weight correlated with the dog’s age, whereas in castrated dogs this relationship could not be established. An atrophic prostate gland was identified histopathologically in 94% of the dogs (16/17).

Castration of male dogs is mainly carried out to decimate overpopulation, which is a problem in many countries [9]. Furthermore, male dogs are often castrated because of undesired behavior [10]. For supressing the sexual hormons in male dogs, castration is a beneficial opportunity [11] and is therefore carried out as adjuvant treatment of prostatic diseases like BPH, prostatic cysts or prostatitis [12–15]. The castration of dogs leads to an atrophy of the glandular structure of the prostate while the stromal components increase [16, 17]. With the help of rectal palpation, a decreased prostatic size 1 week after castration is noticeable [13]. The size of alterations like cysts and abscesses is not reduced after castration as the castration only reduces the amount of prostate tissue, but not the amount of fluid [13]. Castrated dogs have an increased risk of prostatic carcinoma [18]. Additionally, Bryan et al. [19] found out, that castrated dogs have an increased risk for transitional cell carcinoma of the bladder and prostate as well as for adenocarcinoma of the prostate and prostate carcinoma.

Although previous studies evaluated the prostate of intact male dogs in CT-imaging, to the best of our knowledge there are no studies focusing on CT examination of the prostate in castrated dogs.

Since the tissue structure of the prostate gland changes after castration, the attenuation values in CT-images should reflect this change. Accordingly, this study aimed to investigate attenuation values and size of the prostate gland in neutered dogs in pre- and post- contrast CT images. Another purpose of this study was to examine if age-related changes in the prostate can be found in CT-scans of castrated dogs. Furthermore, it was the aim to evaluate if pathological changes in the prostate of castrated dogs are reflected in HU density of the prostate and if differences in the HU between homogenous prostatic tissue and the prostatic tissue with alterations could be found. Additionally, these results should be compared with the results of size and density measurements of the prostate gland in CT scans of intact male dogs of the previous study of Kuhnt et al. [7].

## Results

**Fig. 1:**
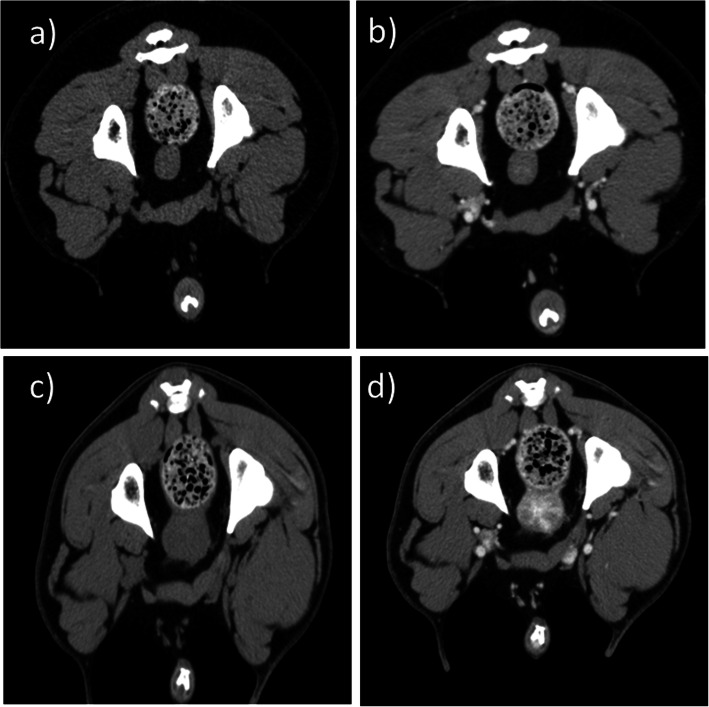
Transverse CT images of the canine prostate. **a** pre-contrast scan of a castrated dog with a homogenous prostate; **b** post-contrast scan of a castrated dog with homogenous prostate; **c** a prostate with alterations in pre-contrast scan of a castrated dog; **d** a prostate with alterations in post-contrast scan of castrated dog

After the CT image evaluation, all dogs (*n* = 36) were classified into two morphological groups. Group 1 (Fig. 1a, b) consisted of dogs with a homogenous prostate (*n* = 25) and dogs that had a prostate with alterations were assigned to Group 2 (*n* = 11) (Fig. 1c, d). Group 1 had a mean age of 8 years (between 1.1 and 13.5 years) mean age of dogs in group 2 was 9.2 years (between 6.6 and 12.3 years). The mean age of dogs in the two morphological groups was not statistically different between the two groups (*p* > 0.05).

**Fig. 2:**
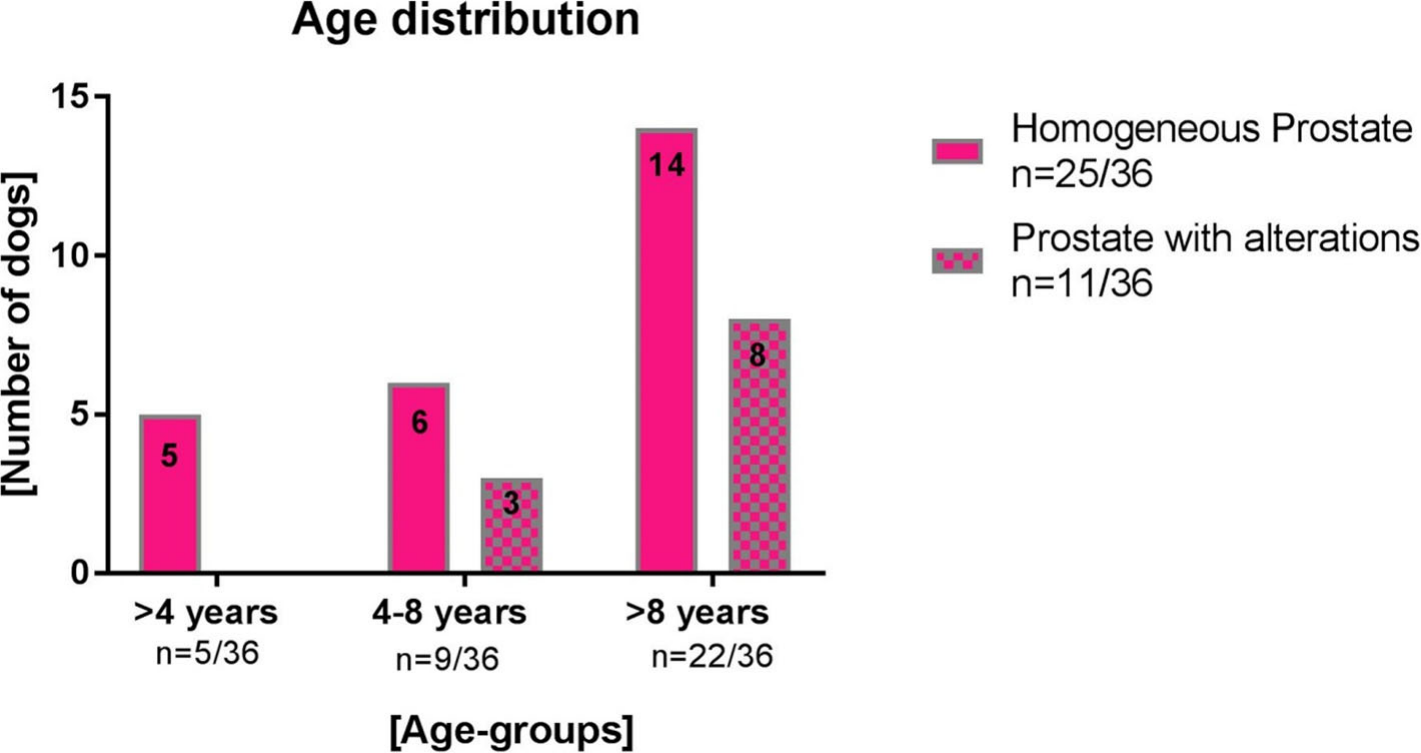
Post-contrast attenuation values (mean HU +/− SD) of homogenous prostates and prostates with alterations of castrated dogs compared with mean values of homogenous prostates, inhomogeneous prostates and prostates with cysts from intact dogs (values derived from Kuhnt et al. [7]. Statistical significance of *p*-value: * (*p* < 0.05), ** (*p* < 0.01), *** (*p* < 0.001)

In addition to the morphological investigation, dogs were assigned to three different age groups (A; B; C) (Fig. 2). In age group A (< 4 years) all dogs had homogenous prostatic tissue (*n* = 5/5). The majority of the dogs in age group B (between 4 and 8 years; *n* = 6/9) and in age group C (> 8 years; 14/22) had alterations as well.

### Attenuation values

The mean attenuation values for of the right and left prostate lobe did not differ statistically from each other, consequently values were summarized. The homogenous prostate gland of castrated dogs was isodens to the pelvic muscles with 43.1 HU (range from to 26.8 to 59.3) in pre-contrast scan CT-scans (Table 1). The altered prostate gland of castrated dogs had a HU of 44.9 (range from 37.2 to 61.9). The pre- and post-contrast attenuation values of each group were statistically different from each other (*p* < 0.05). The attenuation values of castrated dogs with homogenous prostate did not differ statistically significantly (*p* > 0.05) in pre-contrast scans from values measured in inhomogeneous prostates. In the post- contrast images the attenuation values were statistically different between the two morphologic groups (*p* < 0.05). The homogenous prostate gland of castrated dogs in post-contrast scan CT had a mean attenuation value of 77.7 HU (range from 48.4 to 109.7). Mean attenuation value of the altered prostate gland in castrated dogs was 107.4 HU (range from 72.3 to 164.7). Attenuation value data of the two morphological groups is summarized in Table 1.

**Table 1:** Attenuation values of the morphological groups in pre-and post- contrast CT images

Group	Pre- contrast mean [HU] +/− SD	Minimum Value	Maximum value	Post- contrast mean [HU] +/− SD	Minimum Value	Maximum value	p- value between the pre- and post- contrast density
1 (homogenous prostate)	43.1+/−17.8	26.8	59.3	77.7+/−7.0	48.4	109.7	< 0.0001
2 (Prostate with alterations)	44.9+/−31.7	37.2	61.9	107.4+/−7.4	72.3	164.7	< 0.0001

**Fig. 3:**
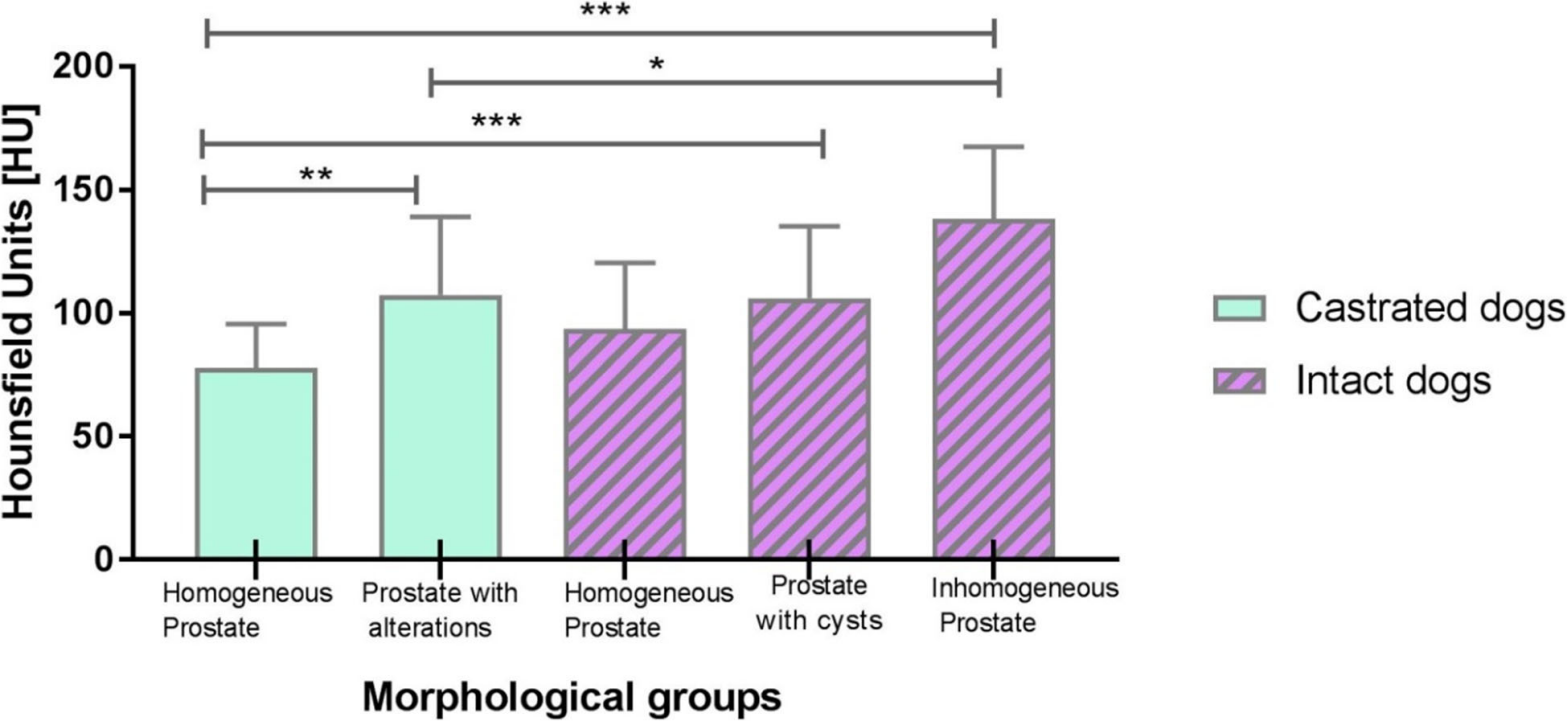
Transverse plan of the CT images of the prostate gland. ROIs were drawn separately in the right and left lobe of the prostate a) homogenous prostate of a castrated dog. b) prostate with alterations of a castrated dog

Furthermore, the attenuation values were compared with the CT attenuation values of intact male dogs investigated in the previous study of Kuhnt et al. [7] (Fig. 3).

There was no statistical difference found for the pre-contrast attenuation values between the groups. The post-contrast attenuation values and the comparison between castrated and intact dogs are shown in (Fig. 3).

Regarding HU measurements no statistically significant differences were found between homogenous prostatic tissue of intact and castrated dogs. Likewise, the attenuation values of prostates from intact dogs with cysts and prostates with pathological changes from castrated dogs did not differ significantly.

Statistically significant differences were found between attenuation values of homogenous prostates from castrated dogs and prostates with alterations from intact dogs (prostates with cysts and inhomogeneous prostates) (*p* < 0.001; *p* < 0.001). Additionally, prostates with alterations from castrated dogs and inhomogeneous prostates from intact dogs showed statistically different attenuation values (*p* < 0.003).

### Prostatic size

The mean height of the prostates from all patients was 1.7 cm (SD (standard deviation) =0.6), the mean width was 1.5 cm (SD = 0.6) and the mean length was 1.7 cm (SD = 0.4). Regarding all patients, the different parameters of the prostatic size [height (H), length (L) and width (W)] are positively and statistically significant correlated with each other (H and W: *r* = 0.8, *p* < 0.0001; H and L: *r* = 0.8, *p* < 0.0001; W and L: *r* = 0.8, *p* < 0.000).

The length, height and width of the prostate and the ratio of the prostatic size did not show statistically significant differences between the homogenous prostate and the prostate with alterations (*p* > 0.05). Prostatic size characteristics of the two morphological dog groups with homogenous prostates and prostates with alterations are summarized in Table 3.

Furthermore, the correlation of the ratio of the prostatic size [ratio height (rH), ratio width (rW), ratio length (rL)] to each other was analysed. Within both groups mean to good correlations were found between following ratios: group 1: rH and rW: *r* = 0.8, *p* < 0.0001; rH and rL: *r* = 0.68, *p* = 0.0002; rW and rL: *r* = 0.73, *p* < 0.0001; group 2: rH and rW: *r* = 0.88, *p* = 0.0003; rH and rL: *r* = 0.6, *p* = 0.05; rW and rL: *r* = 0.85, *p* = 0.0008.

Additionally, no statistically significant correlation was found between the mean age of the dogs in the morphological groups at the time of CT examination and their mean prostatic size (Table 2).

**Table 2:** Pearson’s correlation between age of dogs and prostate dimensions

**Group**		**rH**	**rW**	**rL**
**1(homogenous prostate)**	age (years)	*r* = 0.08 *p* = 0.7	*r* = −0.02 *p* = 0.9	*r* = 0.06 *p* = 0.8
**2 (Prostate with alterations)**	age (years)	*r* = 0.25 *p* = 0.5	*r* = 0.34 *p* = 0.3	*r* = 0.26 *p* = 0.4

*rH* ratio of the height, *rW* ratio of the width, *rL* ratio of the length

When comparing the results of the present study with the prostatic size of the intact male dogs from the study of Kuhnt et al. [7], statistically significant (*p* < 0.05) differences between castrated and intact dogs were found for the ratio of the prostatic width and length (Table 3). All values were smaller in castrated dogs than in intact dogs. The ratio of the height differed significantly (*p* < 0.05) between prostates with homogenous prostatic tissue of castrated dogs and all intact prostates. The ratio of the length, width and height of prostates with alterations from castrated dogs differed significantly from all prostates with alterations (prostates with cysts, inhomogeneous prostates) of intact dogs.

**Table 3:** Representation of the prostate size characteristics of the two morphological groups (homogeneous prostate and prostate with changes) from this study. To facilitate comparisons with values of intact dogs, mean prostate characteristics values of the study by Kuhnt et al. [7] are displayed as groups 3–5

Group	Height (cm)	SDh	Width (cm)	SDw	Length (cm)	SDl	Length 6 LV	SDlv	Age (years)	SDa	rH	SD	rW	SD	rL	SD
Group 1 (neutered, homogenous prostate)	1.5	0.49	1.3	0.47	1.5	0.32	2.5	0.6	8.0	3.7	0.6	0.2	0.5	0.3	0.6	0.1
Group 2 (neutered, prostate with alterations)	2.1	0.68	1.8	0.77	2.0	0.47	2.7	0.4	9.3	1.7	0.7	0.2	0.7	0.2	0.7	0.1
Group 3 (intact, homogenous prostate), from [7]	2.6		3.1		2.6		2.6		5.2		1.0		1.2		1.7	
Group 4 (intact, prostate with cysts), from [7]	4.0		4.1		4.7		2.7		9.2		1.5		1.6		1.7	
Group 5 (intact, inhomogenous prostate), from [7]	3.2		3.4		3.1		2.7		6.4		1.2		1.3		1.2	

*SDh* Standard deviation of the height, *SDw* Standard deviation of the width, *SDlv* Standard deviation of the lumbar vertebra, *SDa* Standard deviation of the age, *rH* ratio of the height, *rW* ratio of the width, *rL* ratio of the length

### Ultrasonographic examination

Most of the dogs were examined using ultrasonography (*n* = 25/36) (Table 4). In 21 (of 25) cases the prostate was considered as homogenous in ultrasound examination, while the other four dogs had alterations. Only 17 of the 21 cases with homogenous prostate (according to ultrasound examination) were judged as homogenous in the CT examination, while four cases showed alterations in CT (Table 4).

**Table 4:**
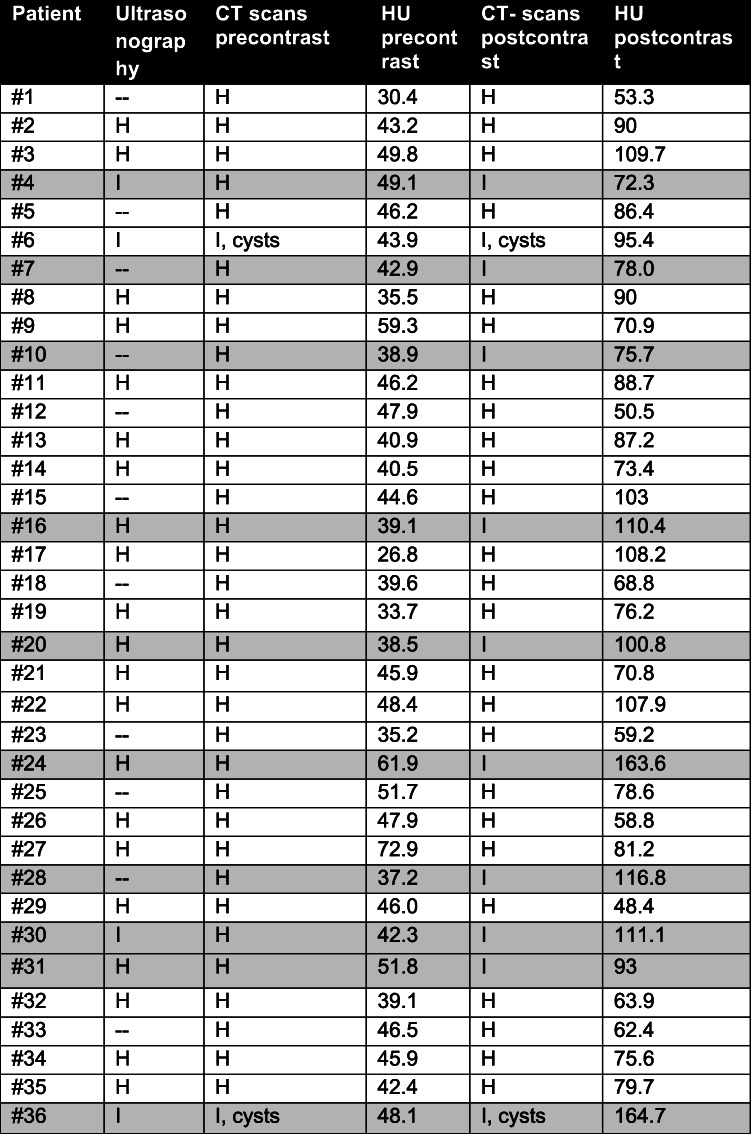
CT examination in comparison to ultrasound examination. Deviating results between the ultrasound and the CT examination were colored grey.

H = Homogenous prostate¸

I = Inhomogeneous prostate

-- = No ultrasound examination available

## Discussion

Most prostatic diseases concern intact male dogs, but also castrated dogs can be affected [20, 21]. However, the evaluation of the prostate of castrated dogs is not easy due to the small prostatic size and the intrapelvic prostatic position of the gland. This may restrict a radiographic examination. The three-dimensional view of the CT [1] helps evaluating the prostate of castrated dogs. To the best of our knowledge, no further studies have evaluated the prostate of castrated dogs in CT, yet. By classifying the patients into three age-groups, the present CT-study showed age-related changes in the prostate structure of neutered and intact dogs. The prostates of young neutered dogs (under 4 years of age) showed a homogenous tissue pattern. Cowan et al. have shown that the prevalence of chronic prostatitis and the presence of bacterial content are reduced in castrated dogs [22]. Thus, the low prevalence of tissue alterations in young neutered dogs could therefore also explain the predominance of a homogeneous prostate tissue pattern in CT imaging in this group of dogs. Another hypothesis for the homogenous pattern of prostate tissue in neutered dogs of under 4 years of age might be that the long-term influence of sex hormones on prostate tissue is low due to castration. In this context, it should be noted that dogs of the young age group underwent castration at different time points. Some dogs were castrated early, others late in the 4 years period. Thus, it’s highly likely that there were different effects on prostatic tissue due to the shorter or longer duration of sex hormones exposal. For non-castrated dogs over 6 years O’Shea showed an increased risk of cysts and BPH [23], which can be visualized by CT as inhomogeneities [7]. In the present study only 33% of the neutered dogs between 4 and 8 years of age showed a prostate with structural changes. Also, in this group the withdrawal of androgens [23] due to castration might explain the predominant homogenous pattern of the prostatic tissue. Unfortunately, the hypothesis about the influence of sex hormone withdrawal on the prostatic tissue appearance in CT could not be proven in the present study because information about the timepoint and reason for castration as well as histopathological analyses were missing.

According to O’Shea et al. intact dogs reach the hyperplastic phase with an age between 6 to 10 years [23]. In this phase the cells can be hypertrophic and electron microscopic evaluation shows an increased glandular volume and decreased prostatic stroma [24]. Furthermore, the hyperplastic phase can result in several cysts or an irregular prostatic pattern [23]. In the study of Kuhnt et al. [7], these changes were displayed in CT examination of intact male dogs in the age group B (4–8 years), where 2/3 of the dogs had an altered prostate pattern in CT-examination. In the oldest age group C (over 8 years), 64% of the castrated dogs had a homogenous prostate. In contrast to this, 84% of the intact male dogs had altered prostates [7]. Thus, intact dogs with an age of 6 years and older have an increased evidence of BPH [25]. A BPH is characterized by an inhomogeneous tissue pattern and cystic alterations [26]. Most of the intact dogs in the oldest age group of the study by Kuhnt et al. [7] had cysts. Accordingly, it can be assumed that the presence of androgens leads to more alterations in the prostate of intact dogs compared to neutered dogs.

It is a limitation of the present study that most of the examined dogs were older than 8 years. Thus, the group size differed across age groups. The high mean age could be explained by the fact that dogs were examined in the CT for age related diseases, such as orthopedic diseases or neoplasia.

Dimitrov et al. [27] described the canine prostatic appearance in CT images as a homogenous, oval, hypodense organ compared to the hyperdense rectal wall. So far, the appearance of the prostate of castrated dogs in the CT was not described in further studies. Therefore, no comparative values are available.

While attenuation values in the two morphological groups did not differ in pre-contrast scans, attenuation values significantly differed in the post contrast CT images. Similar results were found for intact dogs in the study of Pasikowska et al. [6] and in the study of Kuhnt et al. [7]. While both studies found no statistically significant differences between the morphological groups in the pre-contrast attenuation values, both reported significant differences in the post-contrast attenuation values.

Using contrast agent, prostates of neutered dogs which formerly appeared homogenous in pre-contrast scans showed inhomogeneous tissue patterns. This underlines the need of contrast-agent application in imaging the canine prostate gland in CT.

Previous studies concluded that ultrasound is a beneficial tool for evaluating the prostatic size and tissue of intact dogs [8, 28–31]. However, it is mentioned that the beginning of a BPH could sometimes not be diagnosed in the ultrasound examination [32]. The present study indicated that structural changes in the prostate gland of neutered dogs can be seen in contrast CT while in sonographic examination no alterations were detected. It is likely that in castrated dogs pathological changes are easier to be detected in the post contrast CT than in ultrasound inter alia due to the little prostatic size of castrated dogs. Although this finding emphasizes the usefulness of CT as imaging tool, further CT investigations including histopathological analyses are needed to prove the results. Additionally, it has to be mentioned that maybe contrast enhanced ultrasound could also show early alterations in prostatic tissue. In order to find this out, a study has to be conducted with CT examination compared to contrast enhanced ultrasound of the dog’s prostate.

Due to the use of contrast-agent an altered vascularization of the prostatic tissue could be visualized. Only in two cases, an inhomogeneous cystic prostate pattern was found in pre-contrast scans, it was primarily seen in post-contrast CT images. Since alterations like cysts and abscesses are not always reduced after castration [13], inhomogeneities in castrated dogs may be residuals. Inhomogeneous prostates in castrated dogs with a higher amount of enhancement might be indicative for a recently castrated dog. Main limitations of the present study are that the date of castration and the reason for castration were not known. Therefore, it could not be evaluated if more dogs show inhomogeneities shortly after castration. Accordingly, for future studies in this field of research a larger patient collective of dogs with known castration history is indispensable.

One reason for the inhomogeneous appearance of the prostate in post- contrast images could be a prostatic carcinoma. According to Saunders et al. [26] the prostate of affected dogs shows a higher vascularization of the prostate tissue. Regarding the post-contrast prostatic density values of the castrated dogs, the present study found lower values compared to the values of intact dogs in the study of Kuhnt et al. [7]. In canine prostates with hyperplastic tissue, a higher vascularization exists [12]. This may lead to a higher contrast enhancement than in the prostatic tissue of castrated dogs. Unfortunately, the connection between an inhomogeneity or hyperplasia of the prostate due to an increased vascularization could not be proven in our study because no histopathological data were available. Accordingly, a diagnosis based on cytology or histopathology would be of interest and should be included in follow-up studies. Prostatic mineralization and mineralized prostatic cysts are findings which have been reported in dogs suffering from prostatic disease [33, 34]. Bradbury et al. reported that “neutered dogs with prostatic mineralization were very likely to have prostatic neoplasia” [33] and Feeney et al. found that 4/7 dogs with prostate neoplasia had radiographically detectable tissue mineralization [34]. Surprisingly, in the group of 36 dogs examined by us, no mineralization of the prostate was detected. It is likely that these results are due to the very small sample size and the fact that no dog with confirmed prostate neoplasia was among the patient collective. Hypothetically, CT should be well suited to detect tissue mineralization of the prostate at an early stage. Thus, future CT-studies of the canine prostate using a larger group of neutered patients should also investigate the mineralization of the prostate and prove the results by histopathological investigations. A further limitation of the study is that no information about the structure of the prostate gland before castration existed. Further studies are needed, which image the prostate gland before and after castration to describe these image patterns.

Due to the small size of the homogenous prostates in castrated dogs, HUs were measured placing elliptic ROIs over the whole prostate gland. Consequently, the urethra could not be excluded, which may have had an influence on the measured HUs and should be considered as a limitation of the study.

The prostatic size of intact male dogs was examined according to previous research [5–7]. In both morphological groups (homogenous prostate and prostate with alterations) the prostatic ratio parameters were correlated. Consequently, the prostate of castrated dogs seems to grow uniformly, independent of its tissue structure. In the present study the ratio of the prostate size was bigger in the altered prostate than in the homogeneous prostate. These alterations and cysts can be caused by inflammation and ageing processes. After castration the prostatic tissue undergoes atrophy [12, 13, 17, 35] and the prostatic size does not increase with age like in the intact dogs. This atrophy can be also seen in the CT images. By comparison, with higher age the prostate size of castrated dogs does not increase, whereas the prostate size of intact dogs increases.

The ratio of the prostatic height, width and length differed between the prostates of castrated and intact male dogs. In the study of Pasikowska et al. [6] only intact male dogs were included and the ratio of the prostate size in intact dogs with BPH was significantly higher than in dogs with homogenous prostate. The values for the rW, rH and rL of the homogenous prostate in the study of Pasikowska et al. [6] were on average 60% higher than in the present study. Concerning the altered prostate, the values of the intact dogs in the study of Pasikowska et al. [6] were on average 68% higher than in castrated dogs of the present study. It can therefore be said, that the prostate of castrated dogs is significantly smaller than in intact dogs of the study of Pasikowska et al. [6]. In conclusion, the atrophy of the prostate after castration is reflected in the lower post-contrast density as well as in the lesser prostatic size.

## Conclusion

The present study showed that CT is a beneficial diagnostic tool for the evaluation of the prostate gland in castrated male dogs. Reference values for such patients were established including size, attenuation and characteristic tissue structures.

The CT examination enabled to demonstrate differences between the prostate of different age groups and between castrated and intact male dogs. The dog’s prostate shows an atrophy of the acini after castration [36] which was reflected in the higher pre-contrast and in the lower post- contrast density values and the lower prostatic size, in contrast to the values of the intact dogs. The study also demonstrated that the CT could enable a detailed evidence of alterations in the prostatic tissue in contrast to the ultrasound. Therefore, prostatic diseases could be diagnosed with CT, even before they were visible in the ultrasound. It has to be mentioned that the presented results should be considered preliminary until a further study with larger sample size in dogs with well-known castration status and histopathologically confirmed status of the prostates is performed.

## Methods

### Patients

In the present retrospective study CT images of prostates from 36 castrated dogs were evaluated. The dogs were presented in our clinic between July 2009 and November 2015 for abdominal and/ or pelvic diagnostic imaging. The dogs belonged to various breeds and their age ranged between 1.1 years and 13.5 years (mean 8.4 years +/− 13.3 SD). The weight was known in 30 out of 36 dogs and ranged between 3.6 kg and 54 kg (mean 26.8 kg +/− 13.5 SD).

Inclusion criteria were that dogs were castrated and pre- and post-contrast CT images of the prostate and of the 6th lumbar vertebrae were available. However, dogs were excluded when metal streak artifacts from orthopedic implants were seen in the CT data sets.

Dogs were classified into different age groups as well as morphological groups according to the appearance of the prostate gland in contrast and non-contrast CT images by one investigator. This division was similar to the age-groups chosen in the previous study of Kuhnt et al. [7]. Group A included dogs under the age of 4 years. Group B included dogs of an age between 4 and 8 years. Dogs with an age over 8 years belonged to group C.

Depending on the morphologic characteristics of the prostatic tissue, dogs were assigned to two morphological groups. The first group included dogs with homogenous prostatic tissue in the pre- and post-contrast scans. The second group included dogs with structural changes of the prostate gland, like inhomogeneities or cysts. These changes had to be present in pre- or in post-contrast scans.

### CT examination

The CT examination was performed in anesthesia using a 64 multidetector-row CT scanner (Phillips Brilliance 64, Philips GmbH, Hamburg, Germany).

All dogs were anaesthetized with the same protocol: Anesthesia was induced with levomethadon (L-Polamivet 0.2 mg/kg; CP-Pharma Handelsgesellschaft mbH, Burgdorf, Germany), diazepam (Ziapam®, 0.5 mg/kg, Laboratoire TVM, Lempdes, France) and propofol (dose according to effect; Narcofol® CP-Pharma Handelsgesellschaft mbH, Burgdorf, Germany) and maintained with isoflurane (Isofluran CP®, CP-Pharma Handelsgesellschaft mbH, Burgdorf, Germany).

All dogs were examined in dorsal or ventral recumbency according to a previously described protocol [7]. Depending on the dog’s weight the CT scan protocol varied. For dogs with a weight of less than 20 kg the current was 30 mAs and for dogs over 20 kg a current of 200 mAs was used. An automatic tube current modulation function (Phjllips DoseRight-D) adjusted the current during the scan, resulting in different mAs-products for each dog. Independently of the weight, a pitch of 1.171, a slice thickness of 2 mm and a voltage of 120 kVp was used. The field of view was adjusted to a size which included the whole prostate and the 6th lumbar vertebra. All settings were the same for pre- and post-contrast scans. Performing the contrast scan, a non-ionic iodinated contrast agent (2 ml/kg, Xenetix® 300, Guerbet GmbH; Sulzbach, Germany) was injected in the vena cephalica antebrachii or vena saphena lateralis using an injection system (MedRad Vistron CT® 610 System, Indianola USA). The local tracker was positioned in the aorta and the scan started automatically 49 s after reaching 150 HUs.

### CT image analysis

For each dog, CT data were available as transverse image stacks. Sagittal and dorsal images were reconstructed using an image-processing workstation (Extended Brilliance Workspace, Philips Medical Systems, Ohio, USA) and saved in DICOM format with 2 mm slice thickness.

**Fig. 4:**
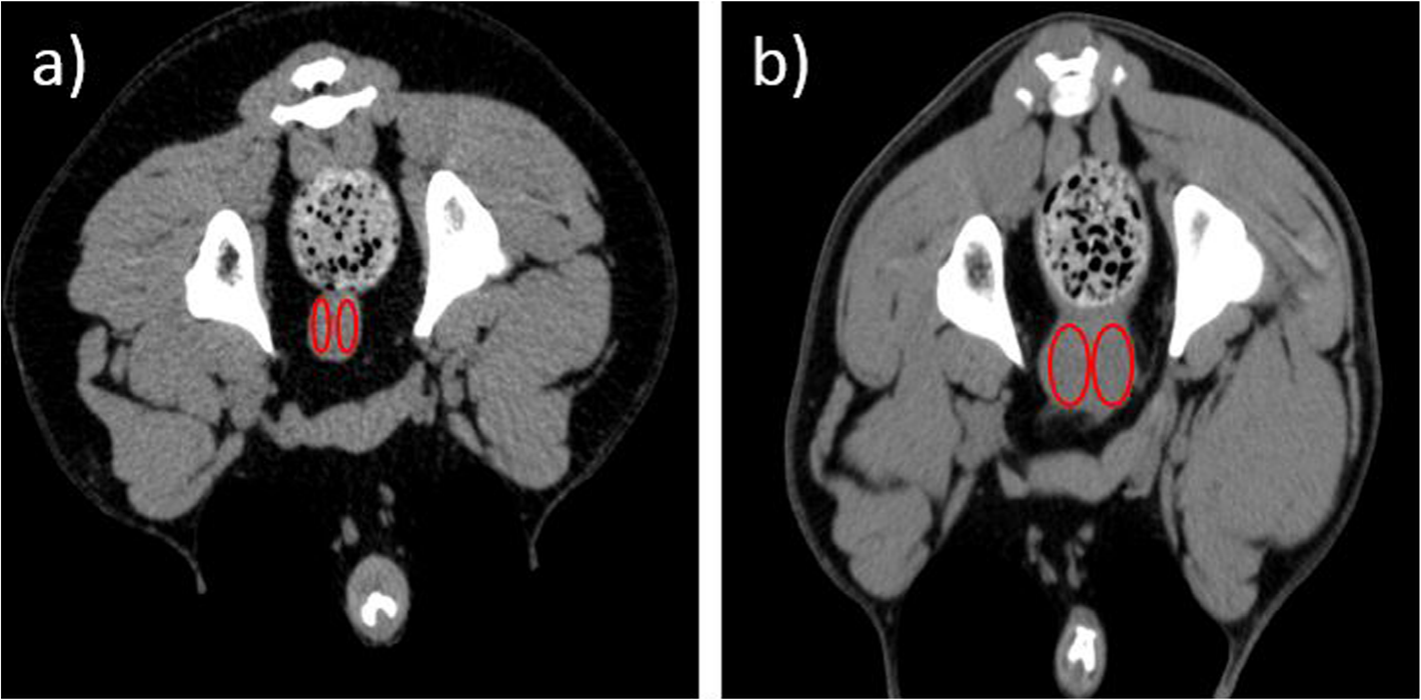
**a**, **b**: CT images demonstrate the measurement of the prostatic size. On the left transverse image (**a**) the red line presents the height, the orange line the width (castrated dog, Magyar Vizsla, 5 years old). On the right image (**b**) the prostatic length is measured (green line) in dorsal plane (castrated male dog, Beagle, 11 years old)

For further analysis DICOM data sets of the prostates were exported to ImageJ (National Institutes of Health, Bethesda, Maryland, USA). Hounsfield Units within the prostate were measured in transverse plane. For HU measurement a circular elliptic region of interest (ROI) was drawn in each prostatic lobe in pre- and post-contrast images using the elliptic ROI tool in ImageJ (Fig. 4a, b). The ROI size was adjusted to touch the outer boundaries of the prostatic lobes and ROIs were set in each slice covering the whole prostatic length. For each prostate lobe median value and standard deviation of the pre- and post- contrast HUs were calculated. The mean HU for every morphological group in pre- and post-contrast images was calculated from the median values of the dogs.

**Fig. 5:**
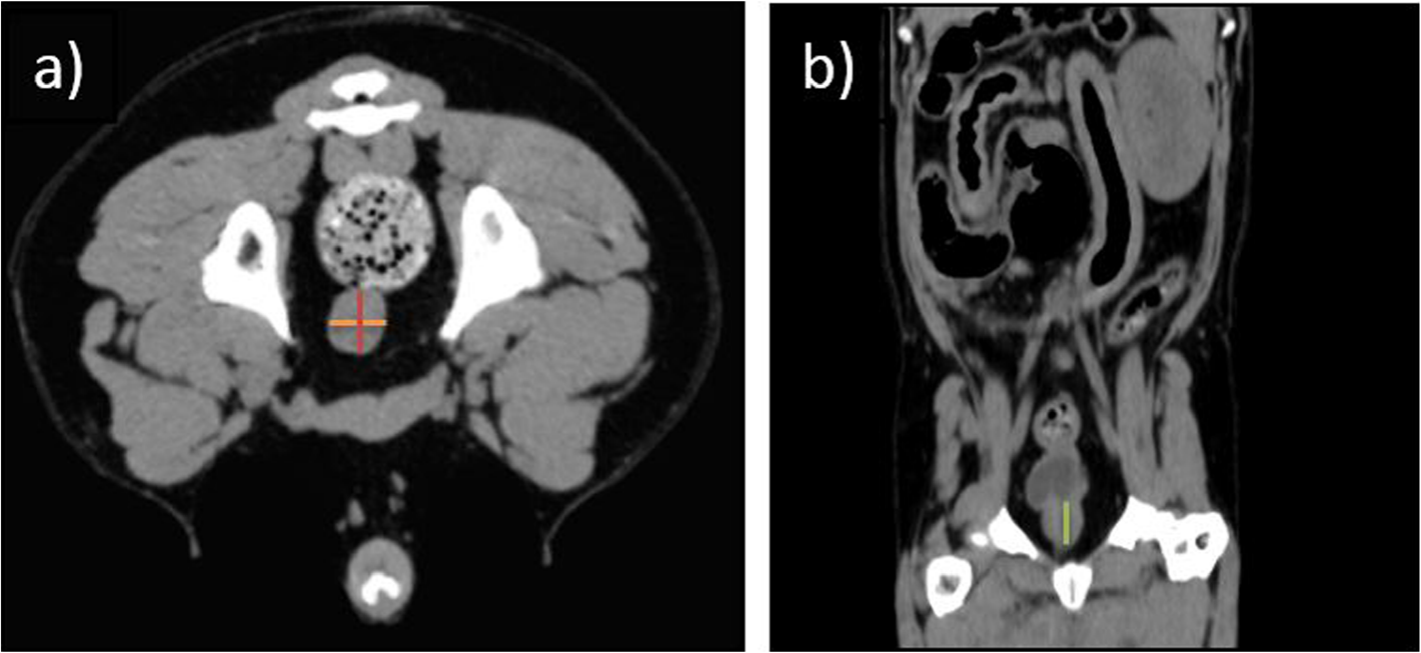
Formula for calculating the ratio of the prostatic height, length and width

For measuring the prostatic size, the method described by Lee et al. [5] was used. On transverse plane the height and width were measured by means of a vertical and horizontal line, drawn through the centrally located intraprostatic part of the urethra (Fig. 5a). The prostatic length was measured on dorsal plane (Fig. 5b).

**Fig. 6:**
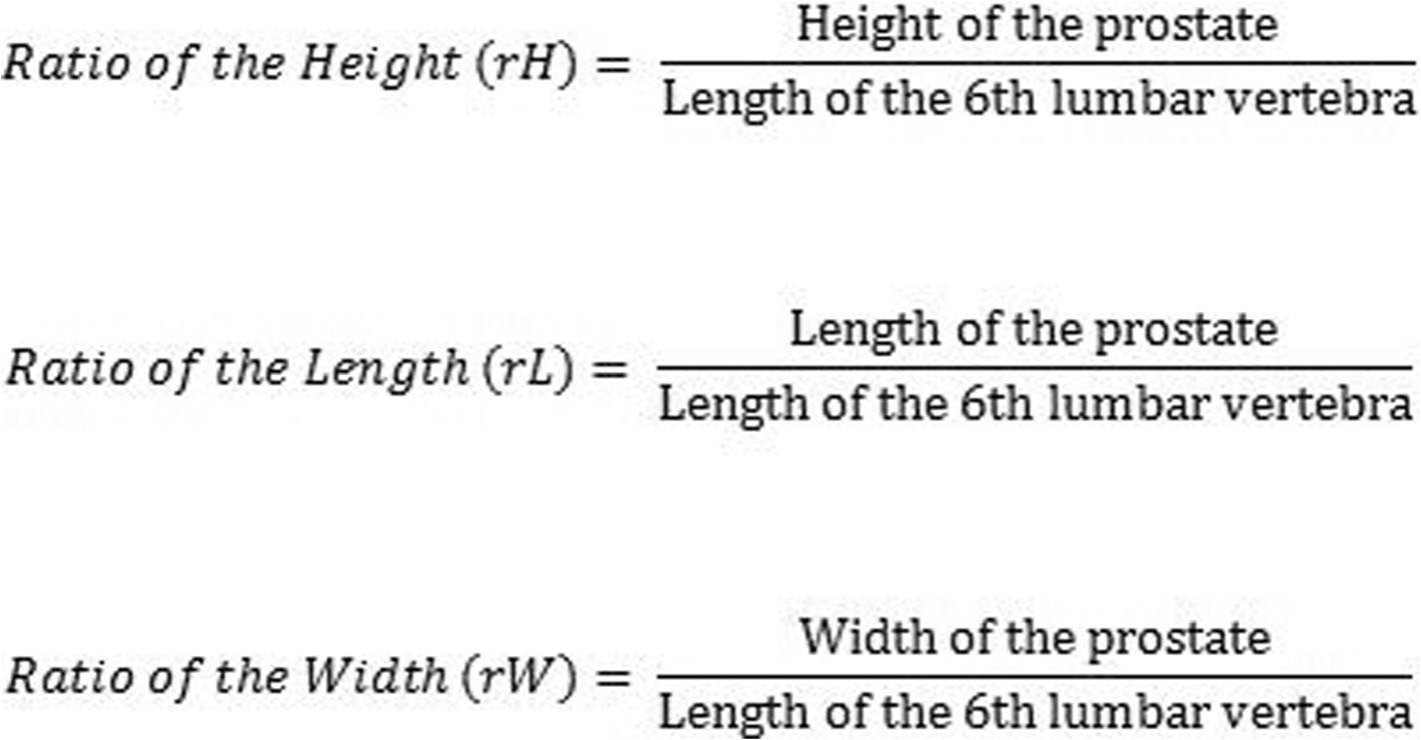
Age-distribution of all dogs, classified according to the morphological appearance of the prostate gland

For the evaluation of the relation between prostatic dimensions and the body size of the dogs, the ratio of the prostatic size to the length of the 6th lumbar vertebra was calculated. Following Lee et al. [5], the present calculated the ratio between height, length and width of the prostate and the length of the 6th lumbar vertebra (Fig. 6).

### Additional examinations

In addition to the CT examination, most dogs underwent an ultrasonographic examination of the prostate (*n* = 25/36). The examination was performed in dorsal recumbency using a B- Mode high-frequency (8 MHz), curvilinear probe and a Logiq 7 GE Healthcare ultrasound device (Wauwatosa, USA).

### Statistical analysis

Statistical analysis was carried out with the SAS® Enterprise Guide® 7.1 (Statistical Analysis Software, Heidelberg, Germany). A paired t-test (Ryan Einot Gabriel Welsch multiple range test) was used for analyzing the statistical differences between the results of the median attenuation values of the two groups. A *p*-value less than 0.05 was considered as statistically significant. The correlation analysis of the prostatic size parameters (height, weight, length) to the patient’s age was carried out by a Pearson correlation. The correlation coefficient r shows the linear relation between two parameters [37]. Correlation was considered weak (*r* = 0–0.5), modest *r* = 0.5–0.8), strong (*r* = 0.8–1) and perfect (*r* = 1) according to the r-values. The statistical significance between the attenuation values of the two morphological groups was analyzed with a factor analysis of variance. The relation between the prostatic dimensions and the morphological groups was examined with a Ryan-Einot-Gabriel-Welsch multiple range-test.

## Data Availability

The datasets used and/or analysed during the current study are available from the corresponding author on reasonable request.

## Abbreviations

BPH: Benign prostatic hyperplasia
CT: Computed tomography
Fig: Figure
H: Height
HU: Hounsfield Units
L: Length
rH: ratio height
rL: ratio length
ROI: Region of interest
rW: ratio width
SD: Standard deviation
W: Width
